# Effect of COVID-19 Curriculum Changes on Medical Student Exam Performance: A Case Series

**DOI:** 10.7759/cureus.58864

**Published:** 2024-04-23

**Authors:** Joshua Ho, Joshua Levy, Nicholas Afshari, Deepal Patel, Shaun Andersen, Edward Simanton, Matthew Linton

**Affiliations:** 1 Medical Education, Kirk Kerkorian School of Medicine at University of Nevada, Las Vegas (UNLV), Las Vegas, USA; 2 Medical Education, Rocky Vista University College of Osteopathic Medicine, Ivins, USA

**Keywords:** medical curriculum, medical education, exam performance, e-learning, online learning, medical school, covid-19

## Abstract

Background: The COVID-19 pandemic caused medical schools to convert to an online format, necessitating a swift change in medical education delivery. New teaching methods were adapted, with some schools having greater success than others. Kirk Kerkorian School of Medicine (KSOM) employed a small-group interactive learning style that consists of eight or fewer medical students and one faculty mentor engaging in group problem-based learning (PBL) twice weekly. This style had clear signs of struggle with a significant decrease in exam performance. Rocky Vista University College of Osteopathic Medicine (RVUCOM) employed a large-group didactic lecture style that consisted of one faculty mentor lecturing hundreds of medical students in a pre-recorded setting five times weekly. This style had greater success with its curriculum adaptation leading to minimal effect on their exam performance. This study aims to investigate whether the type of medical school curriculum (small-group interactive vs. large-group didactic) impacts student exam performance during online learning transitions forced by the COVID-19 pandemic.

Methodology: KSOM and RVUCOM students were grouped into *above-expectations* and *below-expectations* categories based on each institution’s standardized exam performance metrics. Independently sampled t-tests were performed to compare groups. KSOM was classified as a small-group interactive curriculum through its heavy reliance on student-led PBL, whereas RVUCOM was classified as a large-group didactic curriculum through its extensive proctor-led slideshow lectures.

Results: KSOM’s transition to online PBL resulted in fewer students scoring above the national average on the National Board of Medical Examiners (NBME) exams compared to previous cohorts (55% vs. 77%, respectively; *N* = 47 and 78; *P *< 0.01). RVUCOM’s transition to online large-group lectures yielded no significant differences between students who performed *above expectations* and students who performed *below expectations* between their cohorts (63% vs. 65%, respectively; *N *= 305 and 300; *P *> 0.05).

Conclusions: KSOM’s COVID-19 cohort performed significantly worse than RVUCOM’s COVID-19 cohort during their medical school organ-system exams. We believe that the small-group learning at KSOM is less resilient for online curricula compared to the large-group didactics seen at RVUCOM. Understanding which didactic methods can transition to online learning more effectively than others is vital in guiding effective curriculum adjustments as online delivery becomes more prominent.

## Introduction

COVID-19 was initially reported in December 2019 in Wuhan, Hubei Province, China. Characterized by respiratory symptoms similar to pneumonia and acute respiratory distress syndrome (ARDS), COVID-19 was declared a worldwide pandemic on March 11, 2020, by the World Health Organization [1,2]. Consequently, the contagious nature of COVID-19 disrupted medical school curricula and healthcare systems worldwide, forcing medical educators to transition their curricular model to an online format [3]. This change brought upon significant alterations in medical school instruction, with no clear superior method for disseminating information to medical students. Some of the most popular proposed methods of e-learning include scheduling live online video lectures to promote interactive discussions, utilizing small or large breakout rooms to encourage students to participate in active learning activities, and creating self-study pre-recorded online lectures that are made available for medical students at each respective university [1,4,5]. Despite overcoming one of the worst pandemics in history, medical educators must create or identify useful teaching tools that may already exist to efficiently avoid having to transition to e-learning from scratch [6].

Previous research has shown that online learning if implemented well enough can enhance an individual’s satisfaction, motivation, and learning [7-9]. In 2019, a systematic review published by Pei and Wu provided additional evidence in support of online learning being at least as effective as in-person learning for medical students [10]. Another article written by Sriharan in 2020 includes tips for engaging students in virtual classrooms, including curriculum blueprint planning, fostering a comfortable learning environment, and having a knowledgeable faculty facilitator [11]. Predicting the next online curriculum transition may be difficult, but given the rapidly developing nature of technology, educators must determine what tools may be more successful than others at helping medical students achieve success in their careers from an early stage. 

Presumably, many medical schools have internally analyzed student performance as a result of the pandemic, but there is a need for further studies to evaluate the differences between schools. Two studies evaluated first-year medical students’ exam performance and found no significant differences between pre-COVID-19 and COVID-19 cohorts [12,13]. In stark contrast, Andersen et al. noted a significant decrease in their first-year medical students’ exam performance and an overall decline in individuals’ mental health state as a result of the COVID-19 online adaptation [14]. This prompted significant efforts to postulate why students at the Kirk Kerkorian School of Medicine (KSOM) declined academically compared to their peers at other medical schools. 

Students and faculty at Rocky Vista University College of Osteopathic Medicine (RVUCOM) in Ivins, Utah, were also interested in seeing how COVID-19 affected academic performance, thus creating an opportunity for inter-institutional collaboration. Therefore, this study aims to investigate whether the type of medical school curriculum (small-group interactive vs. large-group didactic) impacts student performance during the online learning transition during the COVID-19 pandemic. We will compare exam performance between pre-COVID-19 and COVID-19 cohorts at the two institutions with different curriculum styles to identify factors influencing online learning success.

This research article was presented at the 2023 Western Group for Educational Affairs Conference as an Oral Presentation on April 15, 2023. This article was previously posted on the Research Square preprint server on June 16, 2023. This paper is not pending publication elsewhere.

## Materials and methods

Data collection

At both KSOM and RVUCOM, pre-clinical clerkship directors for Educational Outcomes and Assessment collect medical student examination scores for quality improvement. As such, these test scores were de-identified and obtained from each institution’s respective clerkship director for this study. A waiver for documentation of informed consent was approved by each institution’s IRB approval committee for data usage. All methods were carried out in accordance with relevant guidelines and regulations per the Declaration of Helsinki. All experimental protocols were approved by UNLV and RVUCOM Biomedical IRB committees, respectively. De-identified data for this study were drawn from institutional databases at KSOM and RVUCOM in accordance with an approved IRB protocol for each institution. According to each IRB approval committee, a Waiver for Documentation of Informed Consent was approved on September 20, 2022, by KSOM Biomedical IRB (protocol #UNLV-2022-304). IRB exemption for RVUCOM was approved on October 12, 2022 (protocol RVU IRB #2022-144).

KSOM pre-COVID-19 curriculum 

Before pre-clinical courses begin, students at KSOM participate in an eight-week Immersion, Emergency Response, and Population Health course. This fosters a positive social atmosphere that allows students to interact and form friendships in a low-pressure setting. Additionally, students have the opportunity to learn about the Las Vegas community and its specific challenges, immediately placing students in close-knit communities where they are encouraged to hone their teamwork and peer-to-peer communication. KSOM is a fairly new yet unique medical school that emphasizes small-group student learning throughout its curriculum. Once pre-clinical courses begin, students transition into rotating problem-based learning (PBL) groups where one faculty advisor guides a team of eight or fewer medical students through modified clinical scenarios for each organ system block. At KSOM, PBL is thought to be the highlight of the curriculum where students are expected to gain most of their pre-clinical knowledge, learning from these situations for a total of 6 hours each week. Additionally, students partake in small-group discussions with several clinical faculty in Analytics in Medicine (AIM) and Foundations of Clinical Practice (FCP) courses. For the first 18 months of medical school, students are divided into various small groups each semester to learn epidemiology, biostatistics, medical ethics, and clinical skills/reasoning. Together, these courses promote active participation, enhance critical thinking, and provide opportunities for continual constructive feedback and improvement. Weekly large class lectures are still used to supplement course content and to answer student questions but are not emphasized to be the main learning modality. All PBL and course lectures have mandatory attendance to motivate students to collaborate at in-person facilities. At the end of each three- to four-week course block, students undergo a Custom Standardized National Board of Medical Examiners (NBME) exam where tested content remains consistent across school years.

KSOM COVID-19 curriculum

As COVID-19 reached Las Vegas, KSOM was forced to quickly adopt a new virtual curriculum. Many new, untested changes were introduced into the first-year student courses. For example, the Immersion and Emergency Response Course was essentially postponed for one year until after all the core preclinical courses were completed. Students lost the ability to get to know one another in a low-stakes environment and were immediately thrust into their first core course, not to mention all instruction became virtual. The virtual shift included PBL which was especially challenging for both students and faculty. Because the session was not in-person, students felt less engaged and less inclined to participate in a setting traditionally set up for continuous student interactions. With no in-person lectures, no in-person PBLs, and other peer-to-peer learning opportunities suspended, KSOM lost the core instructional methods that its curriculum was designed around. Breakout rooms were seen as a band-aid solution to the problem, but clearly could not replace the physical small-group learning process.

In a study by Andersen et al., significant differences in academic performance at KSOM between pre-COVID-19 cohorts (*N *= 78) and the COVID-19 cohort (*N *= 47) were found. KSOM students in the pre-COVID-19 and COVID-19 cohorts were recruited for this study using a voluntary response form. Inclusion criteria include KSOM medical students in the pre-COVID-19 and COVID-19 cohorts. Medical students who declined research participation were excluded. To assess exam performances, the authors first analyzed the mean scores of all five exams that the classes of 2022, 2023, and 2024 underwent in their first semester at KSOM. These Custom Standardized NBME exams contain 60 previously used Step 1 questions, and NBME provides an average for how students actually performed on these items during their use of Step 1 in previous years. The NBME national average is provided internally to KSOM’s Director of Educational Outcomes and Assessment. This value is used to determine what performance average is needed for each student to meet current national standards. The custom examinations were built using an equally distributed amount of NBME questions for each organ block to permit individual analysis. Although the true NBME averages for these items are not published publicly, KSOM set a passing threshold for each exam and deemed this threshold to be sufficient in their internal comparison analysis. However, this passing score may not match the NBME average. Therefore, students may be scoring below or above the NBME average and still passing the course. This NBME average was compared to the exam averages that the students at KSOM scored when taking these Customized NBME exams. The performance average of the first five exams of each student was denoted as the NBME-α. Students with a NBME-α score above the provided NBME average were considered as *meeting expectations* and coded as a (1), and students with a NBME-α score below the NBME average were considered as *not meeting expectations* and coded as a (0). Statistical analysis and comparisons between the different cohorts were then performed using independent sampled *t*-tests and* P* < 0.05 was considered significant. Specifically, pre-COVID-19 academic performance was compared to the academic performance of the COVID-19 cohort.

RVUCOM pre-COVID-19 curriculum

The pre-clinical curriculum at RVUCOM is systems-based and begins immediately in the first semester of the first year with a rigorous musculoskeletal unit and accompanying lectures and/or large-group sessions led by academic faculty or practicing clinicians. Before the pandemic, students attended three mandatory classes including an osteopathic principles and practice (OPP) lab, a principles of clinical medicine (PCM) lab, and an in-person anatomy lab. All laboratory sessions were mandatory with students attending in small groups of 8 with faculty preceptors. OPP labs required students to work in small groups that were led by a precepting osteopathic physician, and students received in-depth training on osteopathic diagnosis, treatment, and theory. PCM labs emphasized the fundamentals of medical examination, including history, physical, and other basic doctoring skills, and were also in small groups with a precepting physician, either a DO or MD. Anatomy labs consisted of full cadaveric dissection, with students working in established groups throughout the entire academic year. In contrast, lectures and other large-group sessions were recorded and posted online for students to review at their discretion. Attendance to lectures and large-group sessions was typically not mandatory, resulting in minimal attendance to in-person sessions. This is partly because of RVUCOM’s two-location model (Parker, Colorado, and Ivins, Utah), where all large format sessions originate at one location and are then broadcast to the other location. Written exams (multiple-choice exams) for each organ system block were held in person and on campus in one of the large auditoriums. Practical or competency examinations occurred within lab spaces. 

RVUCOM COVID-19 curriculum

When COVID-19 forced RVUCOM to transition online, administration and faculty members quickly converted all lectures and other large-group sessions to live video streaming (typically Zoom), which were recorded for later review by students. Attendance to the live virtual sessions was typically low and similar to pre-COVID-19 attendance because they did not have mandatory attendance. PCM and OPP lab sessions remained mandatory with COVID-19 protocols being strictly enforced, including required personal protective equipment and contact tracing and isolation for any outbreaks. Anatomy lab dissections transitioned to a virtual format, with dissections completed by faculty or student fellows and streamed live (usually via Zoom) to students. These sessions were mandatory and recorded for later consumption. Importantly, students could walk into faculty offices for any questions, comments, or concerns they had before COVID-19. However, during COVID-19, students were forced to make virtual appointments with faculty, limiting their ability to easily contact professors. All written examinations were completed remotely by students (usually from their homes) and were strictly proctored via a live video feed (usually Zoom). Practical or competency examinations continued to be held within lab spaces, identical to the pre-COVID-19 setting.

RVUCOM does not use Customized Standardized NBME exams; instead, it employs faculty-written examinations. The Assistant Dean of Pre-clinical Education and Curriculum at RVUCOM analyzed and verified that these exams remained consistent across cohorts, even during COVID-19. In contrast to the NBME average used at KSOM to compare means, a standard class average of 83% for each exam was used at RVUCOM to determine if students were meeting expectations. This internally predetermined score of 83% was chosen by the school because it provides a balanced distribution of students who Honor, Pass, or Fail each organ system course. Students at RVUCOM took a total of 22 exams in their first semester and a mean was subsequently calculated from those exam performances. Medical students who had means above 83% were considered to be *meeting expectations* and were coded as a (1). Medical students who had means below 83% were considered *not meeting expectations* and were coded as a (0). Independent sampled t-tests were used for statistical analysis, and *P* < 0.05 was considered significant. Pre-COVID-19 cohort (*N *= 305) academic performance was again compared to the COVID-19 cohort (*N *= 300) academic performance at this institution. RVUCOM students in the pre-COVID-19 and COVID-19 cohorts were recruited for this study using a voluntary response form with identical inclusion and exclusion criteria as KSOM. Notable demographic differences at KSOM and RVUCOM include age, gender, and prior foundational medical knowledge. Attempts were made to match data from each institution, but significant matching could not be fully achieved due to inter-institutional heterogeneity and natural variability among students on both campuses.

## Results

Student exam scores: KSOM

**Figure 1:**
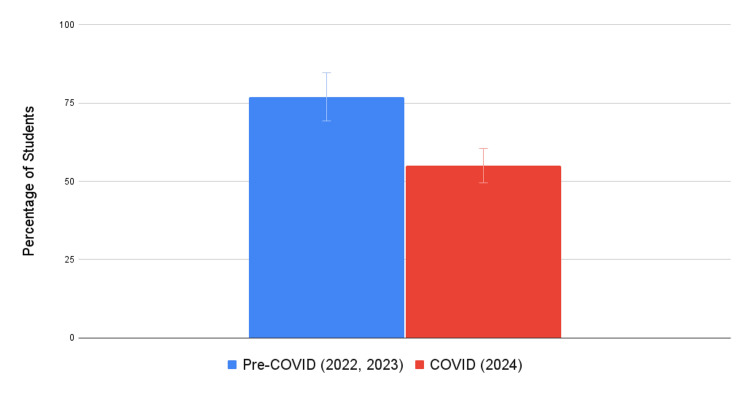
Percentage of KSOM students scoring above expectations during the first semester of the preclinical curriculum. KSOM, Kirk Kerkorian School of Medicine

Student exam scores at KSOM are summarized in Figure 1. At KSOM, the COVID-19 cohort had 55% of students score above the national average compared to 77% of students in the pre-COVID-19 cohorts during their first semester of medical school. These results were statistically significant (*N* = 47, standard deviation [SD] 0.269, versus *N* = 78, SD 0.245; *P* < 0.001). 

Student exam scores: RVUCOM

**Figure 2:**
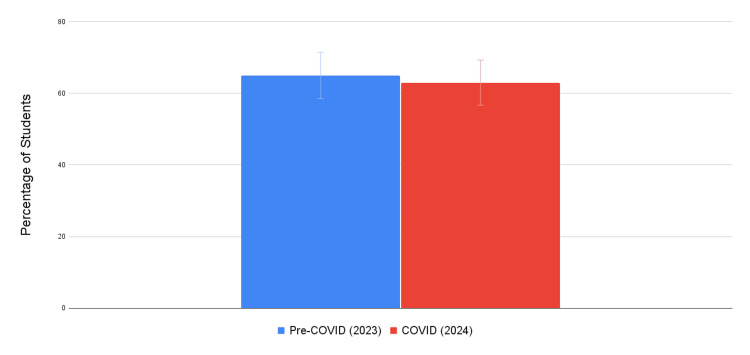
Percentage of RVUCOM students scoring above expectations during the first semester of the preclinical curriculum. RVUCOM, Rocky Vista University College of Osteopathic Medicine

Student exam scores at RVUCOM are summarized in Figure 2 and Table 1. At RVUCOM, the COVID-19 cohort had 63% of students score above their internally determined average of 83% compared to 65% of students in the pre-COVID-19 cohorts during their first semester of medical school. These results were not statistically significant (*N* = 300, SD 5.252, versus *N* = 305, SD 4.863; *P* = 0.282).

**Table 1:** Descriptive statistics of KSOM and RVUCOM student examination scores. RVUCOM, Rocky Vista University College of Osteopathic Medicine; KSOM, Kirk Kerkorian School of Medicine

Examination averages		N	Mean	Standard deviation	*P*-value
KSOM	Pre-COVID-19 cohort	47	77%	0.269	<0.001
	COVID-19 cohort	78	55%	0.245	
RVUCOM	Pre-COVID-19 cohort	305	65%	4.863	0.282
	COVID-19 cohort	300	63%	4.863	

Comparison between schools

Pre-COVID-19 and COVID-19 cohorts from each respective institution were analyzed and grouped based on the number of students performing above or below average. At KSOM, 77% of students scored *above expectations* during pre-COVID-19 compared to 55% of students during COVID-19. At RVUCOM, 65% of students scored *above expectations* during pre-COVID-19 compared to 63% of students during COVID-19. Additionally, Table 2 outlines the two curricula styles used by each respective medical school before transitioning to online education.

**Table 2:** A comparison of curricula styles of Kirk Kerkorian School of Medicine at UNLV and Rocky Vista University College of Medicine (RVUCOM) before COVID-19. UNLV, University of Nevada, Las Vegas; NBME, National Board of Medical Examiners; PBL, problem-based learning; OPP, osteopathic principles and practice; PCM, principles of clinical medicine

	Class size	Attendance requirements	Exam format	Expectation cutoffs	Primary means of education
KSOM	- Cohorts of ~60 students per year	- All lectures and PBL mandatory (~15 hours per week)	- 5 total exams in the first semester - Customized standardized NBME exams	- Averages of five total examinations (NBME-α) compared to NBME national average	- Peer learning groups, PBL, and overview lectures
RVUCOM	- Cohorts of 300 students combined in both campuses	- Large group lectures not mandatory - OPP, PCM, and anatomy labs were mandatory (~6 hours per week)	- 22 total exams in the first semester - Faculty written examinations and laboratory practicals	- Averages of 22 examinations and scoring above 83% cumulatively	- Large-group lectures and small-group laboratory practicals

## Discussion

The results of this study showed significant differences between exam performance at KSOM when comparing COVID-19 and pre-COVID-19 cohorts. However, there were no significant differences between exam performance at RVUCOM when comparing COVID-19 and pre-COVID-19 cohorts.

Delivering medical education through a remote setting is certainly not a novel concept. Over the last decade, several universities have adjusted their curricula to allow for faculty to record and distribute video lectures to medical students [12,15,16]. This allows students to condense their learning at a time, location, and pace that suits them best, something that is becoming increasingly important given the rapidly evolving nature of medical knowledge and information. In 2020, a survey conducted across 39 medical schools demonstrated that flexible learning was perceived to be the most useful benefit of teaching [17]. This is especially crucial to helping medical students who are disabled or may not have the same experiences as other student learners. There are also students whose learning requires additional time or would benefit from recorded teaching material, allowing them to rewind, pause, or replay videos to maximize their learning and retention [18]. Therefore, optimizing an online learning setting for the future would prove beneficial for all parties involved.

KSOM’s transition to online education proved to be problematic for several reasons. According to previous research, a significant number of medical students at this institution dropped below the national average performance on NBME-written exams, along with deteriorating student/faculty relationships and overall mental health during COVID-19 [14]. Additionally, PBL learning was forced to move to online Zoom breakout rooms instead of in-person meetings. Groups of eight individuals with a faculty facilitator met bi-weekly to discuss clinical cases and engage in conversation to learn about the pathophysiology of the disease, practice critical thinking, and communicate effectively. Despite working well in an in-person setting, the online shift resulted in an overwhelming amount of exhaustion or fatigue attributed to the high amount of videoconference involvement, termed *Zoom fatigue* [19]. This exhaustion has also been seen when using other video-calling interface applications such as Google Hangouts, Skype, and FaceTime [20]. However, the unprecedented increase in online meetings due to the pandemic revealed that virtual interactions are extremely difficult to endure for extended periods of time. This was highlighted by Fauville et al. who created a tool to measure fatigue known as the Zoom Exhaustion and Fatigue scale (ZEF) [21]. In addition to the 6 hours of weekly PBL activities, students were also forced to simultaneously participate in mandatory lectures and practical activities, totaling approximately 15 hours of video time. While this amount of instructional time is crucial for fostering learning at the graduate medical level, higher cognitive overload can disrupt and even damage a student’s academic performance, as seen in KSOM students during the pandemic. 

In contrast, the problems faced at KSOM were not prevalent at RVUCOM. During the first semester of classes, RVUCOM medical students were tasked with taking approximately one exam per week for a total of 22 exams. Although this number was significantly higher than the five exams taken at KSOM, students who attended RVUCOM did not have to spend nearly as much time on Zoom lectures as their counterparts. This allowed RVU students to study on their own without having to attend 10+ hours of Zoom lectures, using the resources they found to be most effective. Additionally, when students at RVU watched online lectures, they were structured in a traditional lecture format, allowing students to fully grasp the material being presented to them [22]. In contrast, when KSOM students attended class, they were tasked with learning material in a small group setting where information may be less organized than traditional lectures [22]. We believe that this may have contributed to students performing poorly on exams at KSOM and led to overall negative sentiments regarding PBL’s virtual adaptability. 

PBL and Near Peer Teaching are established and effective means to educate students on medical education [23,24]. Not only do students gain competency in basic sciences, but they also gain valuable professional and clinical skills. However, a large majority of studies on the success of PBL have been based on in-person groups and small discussions. It is suspected that being in person provides the student with the environment necessary to foster professional body language, oral communication, and hands-on teaching methods. Drawing figures, diagrams, and charts on a physical whiteboard allows a student to teach and learn more effectively than other traditional methods [25]. With that being said, this could be a reason that the KSOM COVID-19 cohort did not perform as well academically when compared to their peers. Using Zoom breakout rooms in place of in-person PBL removes that physical aspect that could be important in PBL. The body language and communication skills that are typically fostered in such a setting are lost virtually, especially the important skills of drawing dioramas and charts on a whiteboard. If given another opportunity for e-learning, it may be advisable to limit the amount of small-group learning or necessitate that it takes place in person to facilitate a cohesive experience. If left online, this may negatively impact a student’s ability to retain and apply basic science concepts on their examinations compared to traditional large-group faculty-led teachings. 

A case series examining the various impacts of COVID-19 on medical school curricula is important, but not without its limitations. All educational institutions are protective of their examination methods and scoring, though medical schools are especially under lock and key. This leads to a high degree of difficulty when collaborating as everyone must go through the challenges of de-identifying data, obtaining IRB approval, and disseminating that data to other institutions. One important consequence of this is that the sample size becomes very limited and few schools are willing to collaborate. Future studies should aim to increase the sample size by including more educational institutions with a wide variety of curriculum styles to further investigate the effects of online learning on different curricula.

In addition, confounding bias is present in this study due to the difference in sample sizes between schools and the lack of collection of pre-matriculation data for the different cohorts. As an important note, foundational knowledge already gained by the students before starting the COVID-19 curriculum may differ between the students of the two institutions which would also affect their ability to learn from the modified instructional sessions. Stratifying the students by grade point average, Medical College Admission Test scores, socioeconomic status, studying habits, and other demographic factors may provide more insight into the success of online learning transitions. Our study could be strengthened by performing 2:1 or 3:1 matching using the aforementioned variables to account for differences in sample sizes between institutions.

A future study utilizing a mixed methods approach can help delineate the sources of the differences found in this study. While this study identifies a difference in exam performances between the two institutions, it does not explore the specific reasons why these two curricula have differences. Surveying the students on their satisfaction and motivation within each curriculum can provide insight into the overall *success* of a curriculum.

## Conclusions

In conclusion, this study aimed to investigate the adaptability of KSOM and RVUCOM curricula in response to the COVID-19 pandemic and the transition to a virtual learning environment. Our results suggest that the large-group didactic education model at RVUCOM was more resilient when shifted online compared to the peer-learning education model used at KSOM. There was a significant drop in exam performance for the COVID-19 cohort at KSOM, whereas the RVUCOM COVID-19 cohort had no significant change in their exam performance. As online delivery of educational materials becomes more prominent, it is important to identify which parts of a curriculum can be effectively transitioned to an online setting. This study provides valuable insight into the challenges and successes of online medical education and can drive efforts to improve medical education for new medical students. 
